# Placenta Previa

**Published:** 1876-10

**Authors:** Wm. A. Barry

**Affiliations:** Hot Springs, Ark.


### PLACENTA PREVIA.

BY WM. A. BARRY, M. D., HOT SPRINGS, ARK.

Seeing an interesting article in the June number of your valuable journal, on the subject of Placenta Previa, by Dr. E. Curtis Smith, I deem it proper to report the following case:

Mrs. ———, aged 30, stout and healthy, pregnant with her fourth child, was seized with a hemorrhage on the morning o. the 7th of July, 1876. An examination *per verginam* showed no appreciable dittelation of the os uteri. I gave her small portions of Morphia Sulphas, and the hemorrhage ceased. Gave her the usual charges in regard to taking good care of herself, and instructing to notify me at once if the hemorrhage should return.

On the 6th of August, I was summoned to her again. A much larger hemorrhage had occurred, but had ceased before I reached her. I deemed it prudent not to disturb the patient, and ordering an anodine, left the room.

About two o'clock on the morning of the 14th of August, she was taken, suddenly, with a very profuse uterine hemorrhage. A messenger was dispatched for me in haste, but being absent I did not see the patient until ten o'clock A.M. Found her suffering from a very profuse hemorrhage—pale, fainty and frightened. She was about seven and a half months pregnant. An examination showed the os uteri quite well dilated, and the placenta covering the entire os — a complete Placenta Previa. The woman having never felt the slightest pain, I introduced a tampon composed of lint cotton, and ordered

R. Vinam ergotte,........................................oz. j.
Tincture feri. mar.,....................................dr. j.
Spir. vini gallic, M. S.,...............................oz. ij.

Tablespoonful every half hour. The tampon excited considerable pains before the above mixture could be had. One dose was given, when the pains came on with a vigor, and in half an hour, expelled tampon, placenta and child, and the woman was safe.

The child was still-born, and had the appearance of having been dead for several days.

This case may be interesting in three particulars: First, the uterine contractions, excited by the mechanical iritation of the tampon, as well as its action in checking the hemorrhage. Secondly, the advantage of dry lint cotton over all other substances as a tampon. Thirdly, the increased certainty of the oxytocic action of ergot, when combined with Mur. Tr. Feri.

